# Navigational Bronchoscopy with Cryobiopsy for Diagnosis of ILD

**DOI:** 10.1155/2022/5128432

**Published:** 2022-09-17

**Authors:** Fernando Figueroa Rodriguez, Asfar Ghauri, Tony Abdo, Salim Daouk, Shideler Barbara, Houssein A. Youness

**Affiliations:** Interventional Pulmonary Program, Section of Pulmonary, Critical Care and Sleep Medicine, The Oklahoma City VA Health Care System, USA

## Abstract

**Background:**

Interstitial lung diseases (ILDs) are a group of parenchymal pulmonary diseases in which pathologic diagnosis is essential. Although cryobiopsy has a high diagnostic yield, the complication rate remains high. *Case Presentation*. We report two cases of lung cryobiopsy guided by navigational bronchoscopy (LCB) for the diagnosis of ILD. In both cases, a CT chest angiogram (CTA) using a navigational protocol was performed. Targets were premarked and reached with the navigational system. Radial ultrasound (RU) was applied in combination with fluoroscopy guidance (FG) prior to sampling. Both patients achieved a final diagnosis; they were discharged home after procedure and no complications were noted. *Discussion*. By using a CTA with navigational guidance, we were able to perform cryobiopsy in areas with most disease activity and least vascularization.

**Conclusion:**

LCB used with navigational guidance for the diagnosis of ILD provides may be a safe and effective procedure that provides high diagnostic yield. Limitations include cost, availability, and expertise. Larger trials are needed to confirm the additional benefit.

## 1. Introduction

ILDS are a heterogeneous group of disorders characterized by inflammation and or fibrosis of the lungs that extends beyond the interstitial bed and are classified depending on their radiologic, clinical, and importantly based on their histopathological characteristics.

There are several known etiologies ranging from environmental/occupational exposures and autoimmune diseases to idiopathic. The estimated incidence is 30 per 100000 per year, and the overall prevalence is 80.9 per 100000 per year in males and 67.2 per 100000 per year in females [1, 2].

It is imperative to achieve a confident diagnosis before starting therapy which can include immunosuppression and antifibrotics [2]. In undifferentiated ILD, a pathologic diagnosis is essential. Current sampling techniques include transbronchial bronchoscopic biopsy (TBB) via forceps or cryobiopsy and surgical methods. These procedures pose extremely high risk for either complications, namely, massive airway bleeding (MAB) and/or pneumothorax (PTX) or provide a suboptimal sample. Due to this, it is vital to develop safe techniques with a high yield.

We present two cases of LCB for diagnosis of ILD.

## 2. Case Presentation

### 2.1. Case 1

A 61-year-old man with history of nicotine dependence (40 packs per year) and COPD GOLD 2B was worked up for undifferentiated ILD with progressive functional limitation. Pulmonary function tests with FVCFunctional Vital Capacity (FVC) 63% of predicted, Forced Expiratory Volume in 1 second (FEV1)FEV1 66% of predicted, Total Lung Capacity (TLC) of 59% of predicted, FEV1/FVC of 6883% and and Diffusing Capacity for Carbon Monoxide (DLCO) 61% of predicted, all consistent with a mixed obstructive and restrictive pattern.

**Figure 1:**
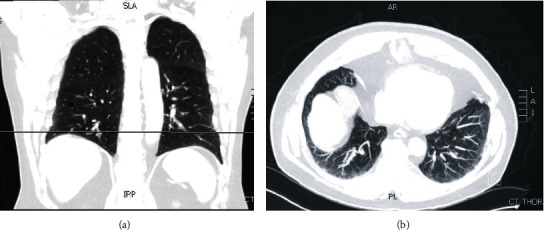
Case 1. (a) CT scan chest coronal view demonstrating subpleural reticulation within the periphery of the lung as well as thin-walled cysts of various sizes (arrows). (b) CT chest using maximum intensity projection protocol demonstrating areas with the most disease activity and least vasculature (circle).

Prior CT chest revealed subpleural reticulation and honeycombing within the periphery of the lung, from the apex to the base. Additionally, thin-walled cysts of various sizes predominantly in the lower lobes were seen (Figure 1). A bronchoscopy with bronchoalveolar lavage (BAL) and EBUS-guided transbronchial needle biopsy obtained from stations 4R, 4L, and 7 were unrevealing. Antinuclear (ANA) and proteinase-3 antibodies were high, but no definitive diagnosis was achieved in two different Multidisciplinary Discussions (MDD), and a recommendation was made to proceed with LCB.

### 2.2. Case 2

A 67-year-old man with history of nicotine dependence (60 packs per year) and COPD GOLD 3B presented with a 6-month history of dyspnea and decrease in exercise tolerance. Pulmonary function Tests with FVC 58%, FEV1 64%, FEV1/FVC of 55% TLC of 66% and DLCO of 54% of predicted, all consistent with a mixed obstructive and restrictive pattern. At initial presentation, a CT of the chest revealed changes consistent with emphysema as well as pulmonary fibrosis with changes suggestive of both usual interstitial pneumonia (UIP) and nonspecific interstitial pneumonia (NSIP). Weakly positive ANA and speckled rheumatoid factor were noted. BAL revealed normal cellularity with macrophage predominance. Imaging was more suggestive of UIP, except for inconsistent cysts which were strongly suggestive of PLCH. MDD Consensus was made to proceed with LCB.

**Figure 2:**
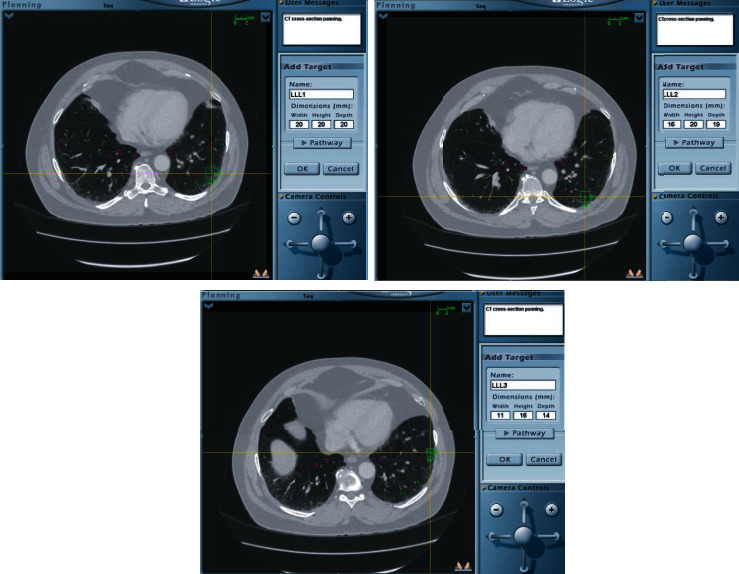
Preprocedure mapping for case 1 using the Medtronic superDimension Navigation System, version 7, Minneapolis, USA. Three targets were premarked in the left lower lobe (LLL).

**Figure 3:**
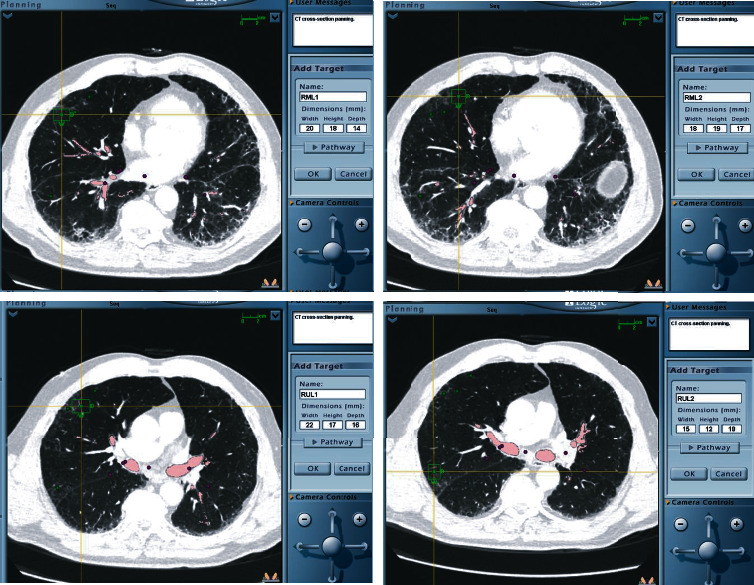
Preprocedure mapping for case 2 using the Medtronic superDimension Navigation System, version 7, Minneapolis, USA. Two targets were premarked in RML and two in the right upper lobe (RUL).

**Figure 4:**
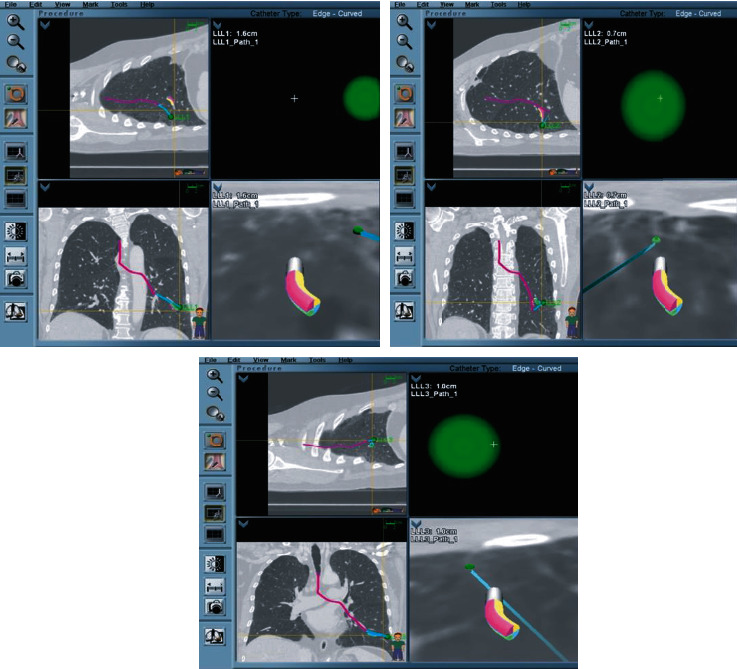
Navigational bronchoscopy on case one. All target areas were reached with the ability to virtually visualize the proximity to the pleural line.

**Figure 5:**
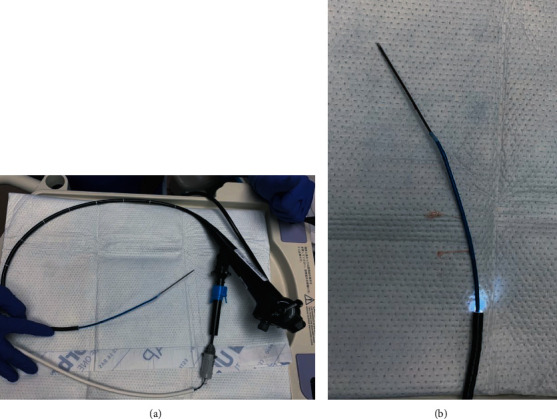
Equipment used for the TBC navigational bronchoscopy. (a) Proximal to distal: Olympus therapeutic bronchoscope, Edge™ extended working channel, flexible single use 1.7 mm cryoprobe catheter (ERBECRYO® 2).

In both cases, a CTA using a navigational protocol (slice thickness and interval of 1 mm) was performed to target the site with the least vascularization and most disease activity. Preprocedure mapping was performed using the Medtronic superDimension Navigation System, version 7. Three targets were premarked in the left lower lobe (LLL) for case 1 (Figure 2) and four targets in case 2 (two in the right upper lobe and two in right middle lobe) (Figure 3); all of them were at one cm from the pleura. The patients were intubated with a size 8.0 cm endotracheal tube (ETT). An endobronchial blocker (EBB) was placed in parallel, outside the ETT, and was advanced under direct bronchoscopic vision into the target bronchus. The Edge™ locatable guide was placed over the 180-degree Edge™ extended working channel; the system was advanced through the bronchoscope. Airway mapping with automated registration was completed. All targets were reached with the Edge™ navigational catheter (Figure 4). The navigational sheath was left in place, and the absence of pulsating blood vessels was confirmed with radial ultrasound. Under fluoroscopic guidance, the 1.7 mm probe (ERBECRYO® 2) was advanced into the sheath until the pleura was reached and then retracted one cm. A trial of freezing the 1.7 mm probe in sterile water was done prior to the bronchoscopy. Four-second freezing time provided us with the desired sample biopsy of 5 mm; as such, a four-second freeze time was used to acquire all biopsies. For case 1, the sample was 3.79 × 3.37 mm, whereas for case two, it was 3.8 × 3.11 mm. The system including the sheath, the cryoprobe, and the bronchoscope was pulled en block (Figure 5). The EBB was inflated for two minutes prophylactically after every specimen, no bleeding was noted upon deflation of the EBB balloon in both cases.

During the entire procedure, the anesthesia and the pulmonary team oversaw the monitoring vital signs including respiratory, heart rate, blood pressure, temperature, oxygen saturation, peak, and plateau pressure. Bleeding was monitored in real time via bronchoscopy. At the end of the procedure, a bedside lung ultrasound as well as a chest X-ray were performed to rule out pneumothorax.

## 3. Discussion

Despite the involvement of different specialists in MDD, histopathology remains imperative for undifferentiated ILD diagnosis [1]. Current techniques providing adequate samples include surgical lung biopsy and TBB [2, 3]. For elective procedures, severe complications were reported in 8% and 4%, while death occurred in 1.7% and 0% after open surgical lung biopsy and video-assisted thoracoscopic surgery (VATS), respectively [4, 5]. VATS remains the gold standard for tissue diagnosis; nonetheless, a significant number of patients may not be referred or elect not to undergo the procedure [4, 5].

TBCB was shown to be able to assist in diagnosing ILD, with two main complications: pneumothorax (PTX) and major airway bleed (MAB). TBB is usually done by advancing the cryoprobe through the bronchoscope under Fluoroscopic Guidance (FG). Once the pleura is reached, the probe is pulled back one cm before cryofreezing. FG is however of limited value when the target location is close to the anterior or posterior chest wall as compared to the lateral aspect. In such a scenario, the operator relies on increased resistance to advancing the cryoprobe as a surrogate of reaching the pleura. However, the inability to advance the cryoprobe further can occur in the setting of a blocked airway and, assuming that the pleural was reached, can lead to MAB as a result of sampling of central vasculature; this is seen in up to 16% of patients with almost 5% experiencing life-threatening bleeding [4]. When the target site is within 1 cm of the pleura, PTX can occur with a reported rate of 14 to 36.6% [6].

The recent introduction of a longer flexible single-use cryoprobe catheter (ERBECRYO® 2) of 1.15 m length allowed the use of that catheter through the Medtronic Super Dimension Navigation System. As such, we were able to reach premarked targets located at 1 cm from the pleura. This adds a layer of safety as we were able to visualize the tip of the probe and the virtual pleural line distally while avoiding central vasculature.

As for the yield, the interventionalist chooses the lobe to biopsy based on prior CT images with the ability to control the path of the cryoprobe during bronchoscopy. Previous studies showed that radial endobronchial ultrasound (RUS) can be used for assessing the biopsy site before the cryobiopsy [7]. Dense signaling on RUS was shown to correlate with consolidation on HRCT [8]. A yield of 80% was seen with the use of R-EBUS in 34 patients undergoing TBB [9]. However, the path taken by the cryoprobe may be different than that taken by the radial probe. In addition, the operator has limited ability to navigate the airway tree to reach an optimal and safe location to biopsy. By preplanning with the CT angiogram navigational protocol, we were able to select areas to the sample that have the highest disease activity and least amount of vascularization peripherally. By using the navigational sheath with the locatable guide, we were able to reach all preplanned targets; the RU evaluation occurred through the sheath and thus provided a real-time assessment at the location of the biopsy.

Changes in lung anatomy between the preprocedural CT scan and the bronchoscopy are a limitation, like atelectasis, which can lead to a divergence between the expected and the actual location of the marked target [10]. Cone beam CT scan (CBCT) guidance has been shown to reduce this effect when used to guide lung cryobiopsy resulting in high diagnostic yield with no periprocedural complications [11]. CBCT is not currently readily available when compared to bronchoscopic navigational systems.

Additional limitations of this technique include the cost of navigational bronchoscopy, availability, and expertise. However, the potential of improving the yield and safety of transbronchial cryobiopsy may justify the additional costs. Further studies are needed to answer this question.

## 4. Follow-Up

**Figure 6:**
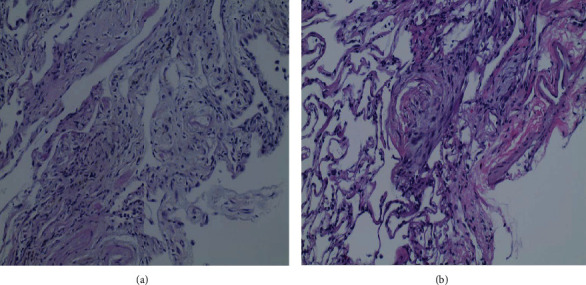
(a) Case 1. Patchy interstitial fibrosis, pigmented macrophages, lymphocytes, and rare eosinophils consistent with PLCH. (b) Fibromyxoid foci (Masson bodies) and fibrosis with sparse lymphoid infiltrate compatible with HP.

The final pathology report was consistent with pulmonary Langerhans cell histiocytosis in case 1 (Figure 6(a)). For case 2, the pathology report was highly suggestive of chronic HP, which was the final diagnosis per the MDT (Figure 6(b)). Both patients were discharged home the same day with no subsequent complications.
